# Composition of place: towards a compositional view of functional space

**DOI:** 10.1080/15230406.2019.1598894

**Published:** 2019-06-06

**Authors:** Emmanuel Papadakis, Bernd Resch, Thomas Blaschke

**Affiliations:** Department of Geoinformatics - Z_GIS, University of Salzburg, Salzburg, Austria

**Keywords:** Place, rule-based, components, place-based GIS, function-based query

## Abstract

A long-standing question in GIScience is whether geographic information systems (GIS) facilitates an adequate quantifiable representation of the concept of place. Considering the difficulties of quantifying elusive concepts related to place, several researchers focus on more tangible dimensions of the human understanding of place. The most common approaches are semantic enrichment of spatial information and holistic conceptualization of the notion of place. However, these approaches give emphasis on either space or human meaning, or they mainly exist as concepts without practically proven usable artifacts. A partial answer to this problem was proposed by the function-based model that treats place as functional space. This paper focuses primarily on the level of composition, describing and formalizing it as a rule-based framework with the following objectives: (a) contribute to the formalization efforts of the notion of place and its integration within GIS and (b) maintain tangible properties intertwined with the human understanding of place. The operationalization potential of the proposed framework is illustrated with an example of identifying the shopping areas in an urban region. The results show that the proposed model is able to capture all shopping malls as well as other areas that are not explicitly labeled as such but still function similarly to a shopping mall.

## 1. Introduction

Geographic information systems (GIS) use various forms of data models to represent the geographic world. Within the basic structures of spatial representation, that is, vector and raster (Frank, 1992b), each method results in a geometrical view of space that conforms to mathematical and cartographic principles. People, on the other hand, perceive and understand the geographic world through the experience gained by interacting with the spatial environment. Place may thus be seen as experiential space from a behavioral standpoint (Couclelis, 1992; Graus, 2017). For Curry (1996), “place is a human invention to describe space”, while, from a humanistic perspective, Tuan (1979) talks of place as “space infused with human meaning”. A long-standing question is whether the capabilities of GIS can enable adequate quantifiable representations of such an elusive concept, so as to truly do justice to the term “place-based GIS”.

Considering the philosophical difficulties of quantifying notions such as “human meaning” or “human experience”, researchers interested in describing and implementing a place-based GIS have tended to focus on the more tangible dimensions that may be ascribed to people’s understanding of place including place name and type, semantic descriptions, afforded activities, or even semantic relations between physical entities and places. We acknowledge the importance of the emotional riches that characterize people’s attachments, memories, experiences, and feelings about places. However, this paper is dedicated to examining the challenge of integrating place within GIS; therefore, we argue that “place” can be at least partially defined by the aforementioned tangible dimensions, due to their analytic and formalizable nature. While this view may be partial, it still is significant since it can capture the way people interact with space transforming it into place. In the remainder of this document, any use of the term “place” refers to this partial but practical view of place. In particular, using as a basis the essential kinds of place inference (Scheider & Janowicz, 2014, p. 101), we assume that in the context of place-based GIS, a formalizable view of place should be able to address questions about the following aspects:

- **Place identification/classification**: features that indicate whether something is a place. These features include identifiers and classifiers, which point out features that make a place differ or be similar to other places (introducing types of places), respectively.
- **Place equipment**: entities associated with a place. They range from simple objects with properties to complex entities, such as places included in other places.
- **Place affordances**: human actions afforded by a place. Associating affordances with places converts the latter into primary objects of human interest.
- **Place localization**: relative or absolute location of a place on space. The temporal dimension plays an essential role since places are created, cease to exist, or may even move in space.

Several works in the literature attempt to formalize place by focusing on one (or more) of the aforementioned aspects. They range from semantic enrichment of spatial information (e.g. digital gazetteers (Goodchild & Hill, 2008)), or associating spatial objects with qualitative spatial relations (Frank, 1992a) to more complex solutions, such as cognitive representation of space (e.g. mental maps (Sanders & Porter, 2010)), knowledge graphs (Vasardani, Timpf, Winter, & Tomko, 2013), or hierarchies of affordances (Jordan, Raubal, Gartrell, & Egenhofer, 1998). The majority of the existing approaches focuses on representing place from either a spatial or a semantic perspective in an inversely proportional manner. On the one hand, there are methods that treat place as a spatial object augmented with trivial or limited semantics; in this way, they obscure several key elements that associate place with human understanding, such as interaction or activities. On the other hand, there are methods based on sophisticated models which provide sufficient information on how the knowledge about places is structured and reasoned with. The downside, however, is that many of these methods exist only as concepts or they are mainly applied in the domain of knowledge representation. The spatial information provided in this case can be overwhelmingly subjective and difficult to be projected using GIS (e.g. derived by human opinions), or too simplistic (e.g. attributed points) that do not capture the complexity of the associated semantics of the place under question.

A step towards addressing the aforementioned limitations is the function-based model of place in the work of Papadakis, Resch, and Blaschke (2016). Instead of affordances, the model assumes that place is space associated with particular functions and that appropriate forms of functionality support a place’s ability to satisfy specific human desires and intentions. Whereas the concept of affordance is *holistic*, that of function is *analytic*, explaining “how” rather than “what” is supported. This is beneficial for the purposes of this work since it facilitates formalization and integration into GIS. The full model integrates five main levels of semantic resolution: purpose, function, composition, components, and data. These levels originate from the concept of the object of discourse as described by Couclelis (2010). Since place takes part in human discourse, it can be described by the sequence of the aforementioned levels reflecting a top-down process of projecting (practical) human intentions on space, down to the data required for appropriate quantification.

The present paper proposes the formalization of place using parts of the aforementioned model, focusing in particular on the relationship between function, spatial composition, and components: it regards place as a system of spatially organized components that enable a particular functionality. Components are considered as physical entities that enable or disable particular functions and eventually build a place. This approach facilitates an inward inspection of place, allowing causal justification of its thematic information, while avoiding limitations granted by representations based on simple word structures or semantically infused spatial objects. The identity of places is derived from how a place operates instead of how a place is perceived (as it happens with affordances), which provides an adequate level of intersubjectivity. We employ a rule-based approach, expressed as set theory combined with first-order logic, to develop instances of place compositions that enable the functions under question.

The objective of this paper is to contribute to the efforts towards the formalization of the notion of place and its integration within digital systems, and GIS in particular. It also seeks to connect with the substantive literature on place as approached from the angles of affordances, human interactions with the spatial environment, and other intention-oriented perspectives on space. The proposed model aims to address the following indicative questions/objectives:

- Which components allow or forbid the development of a function?
- What is the required thematic information and spatial organization of a set of components that would allow a place to offer particular functions?
- How can the functional view of a place be formally represented and integrated into a GIS?
- How can places be identified using their offered functions?
- How can places be classified based on their functionality?
- How can places be located based on their offered functions?
- What is the approximated spatial extent of a place based on its supported functions?

The remainder of this paper is organized as follows. Section 2 provides an overview of approaches that focus on the quantitative representation of place, especially those that associate place with human goals and spatial behavior. Section 3 focuses on the definition and formalization of the level of composition of place, as introduced in the function-based model of place. Section 4 offers an illustrative example of a place-based query for identifying the shopping areas in an urban environment, followed by the evaluation of the results and a brief discussion and reflection on the advantages and limitations of the proposed approach. We then offer some concluding remarks along with directions for future work.

## 2. Background and related work

This section lists the most widely established approaches of formalizing place while enabling its quantification and integration within GIS. As mentioned in Section 1, we mainly focus on works that deal with the more tangible dimensions of people’s understanding of place.

Ontologies (Fensel, 2001) form one of the most common ways of organizing and representing knowledge, ranging from general to specific domains. In the case of the notion of place, there are several developed instances that may be exclusively specialized on the topic such as geonames (http://www.geonames.org/ontology) or general ones that describe place as a part of an upper level ontology such as schema (https://schema.org/Place). Formalizations like these are usually extended with linked data methods that allow the knowledge to be digitized, transferred, and interpreted by computers. Although ontology-based approaches provide details that foster a deeper understanding of place, they include spatial information that is either vague, qualitative, and associated with other landmarks (e.g. placenames and relative locations) or does not conform to human perception (e.g. administrative regions and coordinates).

Ontological gazetteers (Janowicz & Keßler, 2008) extend the simplistic format of digital gazetteers (Goodchild & Hill, 2008) with knowledge graphs, which introduce thematic information about places of interest such as temporal information, parthood relations, and other semantics that outline the context of the place under consideration. However, the attached semantics primarily refer to simple thematic properties rather than formalizing place as a full concept. For instance, they do not include sufficient information on how people interact with place. In addition, the spatial representation provided by ontological gazetteers has the form of geometric entities (e.g. points, lines, or polygons) with fiat boundaries (Smith, 2001). Such representations exist in the mathematical plane and do not converge with human understanding or perception. Further technical limitations include domain dependence and place identity: different knowledge graphs are derived from the needs of different domains. Consequently, gazetteers with different schemas may include entries for the same place, which may lead to confusion and obscure identity matching.

Several works rely on the simplistic formalization of place introduced by gazetteers. Vögele, Schlieder, and Visser (2003) focus on organizing geographic regions identified by place names based on their thematic and spatial relevance. In particular, they extend a graph-based organization of related place names by adding new ones which are related to the existing through containment or parthood. An alternative approach is the extension of place names with features of spatial cognition, such as the principle of contrast: “People conceptualize a portion of space as place if it shows wholeness against its environment” (Winter & Freksa, 2012). Considering that places are containers of objects and events, it is possible to answer localization queries by identifying locations equipped with elements that make them differ from their surroundings. Alazzawi, Abdelmoty, and Jones (2012) focus on methods for mining knowledge about activities and services that are commonly associated with place types. They produce a schema that associates place types with offered service types and affordances, based on the co-occurrence of language patterns retrieved from geographically focused resources.

All of the aforementioned works rely on place names; hence, they face the same challenges as gazetteers which introduced this formalization. Specifically, human activities or containment of objects are completely excluded from the notion of place. Also, with the exception of Vögele et al. (2003), vector representations are not considered, which hinders integration within GIS.

The concept of semantic place (Scheider & Purves, 2013) augments the ontological formalization of place with relational semantics derived from text corpora. Consequently, the knowledge about places comprises not only properties but also relations between the latter and implicit places or other entities. This formalization conforms to the human perception of space using objects and relations (Couclelis, 1992; Tversky, 2003). It also addresses the inability of places to be bounded within precise reference systems, as opposed to gazetteers (Goodchild & Hill, 2008) and facilitates formalization of complex places whose description is not possible with simple word lists. However, it mainly addresses problems related to place localization, while the spatial extent and intrinsic characteristics of place are neglected.

Scheider and Janowicz treat place as an entity that is associated with the activities and actions that are afforded by the objects contained within (Jordan et al., 1998; Kuhn, 2001). Based on this definition, they introduce place reference systems which are independent of spatial coordinates. Simulation is used to determine whether a potential activity is afforded by a place. Places are then localized based on the locations of the objects involved in the activity or activities afforded by that place. This work heavily depends on human perception, which may compromise the objectivity of results. Also, the work remains at a theoretical level; the complexity of cognitive simulations may raise important obstacles when it comes to implementation. Moreover, containment is a key factor in place reference systems; however, relational semantics are not addressed properly; for instance, the activity of walking cannot be afforded by a place containing a path and a highway, when these intersect with each other.

Cognitive regions (Montello, Friedman, & Phillips, 2014) are vague regions frequently used in the vernacular in order to ask questions about localization. They represent concepts that are widely acceptable from groups of people, such as the “city center” (Montello, Goodchild, Gottsegen, & Fohl, 2003). Montello et al. (2014) argue that cognitive regions form a super set of that of the concept of place; thus, it should be expected that delineating cognitive regions present similar challenges to delineating and locating places. A data-driven approach to delineate and semantically enrich cognitive regions is conducted by Gao et al. (2017) relying on various data sources ranging from social media to blogs and encyclopedias. Particularly, thematic information about a particular type of cognitive region (i.e. North and South California) is derived from topic modeling algorithms. Then, the extracted information is extended with spatial properties by utilizing data mining techniques and fuzzy membership methods. The end result, adjusted by probabilistic models, introduces a fixed grid that indicates the likelihood that each cell is of the same type as the cognitive region in question. A relevant data-driven approach is presented by Gao, Janowicz, and Couclelis (2017), with the authors using probabilistic models to describe the semantic signature of functional regions. These regions can be considered as places found on space that support certain types of services. The list of possible services is derived from the venue categorization provided by the Foursquare platform (https://developer.foursquare.com/docs/resources/categories). In particular, the functional context of a delineated region is extracted based on the co-occurrence patterns of the types of the included points of interest. As a result, a region is attributed with a particular functionality, expressed by weighted key words (i.e. 90% shopping, 10% nightlife).

Utilizing probabilities and data-driven methods while describing places allows the vagueness of human perception of space to be confined. However, the bottom-up nature of the last two approaches does not sufficiently account for the human understanding of place; instead, it indicates the association of particular space footprints with human-generated data, such as activities, opinions, and others. The extracted information is exclusively data-dependent, as it is not framed by any model. Furthermore, the assigned probabilities express the statistical significance derived by unsupervised data analysis, which is not necessarily comprehensible from a human perspective.

To summarize, works that rely on place names and semantics are limited to a simplistic view of place; more elaborate models are either purely theoretical or highly subjective and lack adequate spatial projections, whereas data-driven approaches are not easily interpretable by humans. To address these limitations, the proposed methodology adopts a composite view of place. According to this view, a place is treated as neither a semantically enriched spatial object nor a collection of spatially projected thematic data. Instead, it is represented as a dynamically constructed entity, whose components, along with their spatial organization, reveal its spatial extent, while at the same time capturing the more tangible dimensions ascribed to people’s understanding of place. For instance, a place that offers shopping opportunities is delineated by (the surrounding area of) the comprising shops. Moreover, as shown in Section 4, the adopted rule-based formalization is directly implementable, facilitating its integration into GIS.

## 3. Methodology

As mentioned in Section 1, the function-based model of place (Papadakis et al., 2016) is the foundation of our work. This model assumes that people interact with places through the functions that they provide, in order to satisfy potential purposes. According to Papadakis et al. (2016), place is space associated with particular functionality and is described with five consecutive levels of semantic resolution: purpose, function, composition, components, and data. These levels reflect a top-down process of how human intentions can be projected on space (Couclelis, 2010, pp. 1796–1799) infusing it with elements of human experience, such as interaction and eventually converting it to place. In particular, a place satisfies a human purpose by offering a set of functions. These functions permit or prevent human interaction with the place, hence, facilitating or obstructing the initial purpose to be satisfied. The key elements that enable the required functions are spatial organization and properties of the components that are essential for the individual functions. Finally, components can be broken down into spatial and semantic properties, which eventually can provide a representation of place as rigid data.

Considering the above, this section proposes a new formalization of place based on the level of composition and its association with the adjacent levels of functions and components. This approach conforms to synthetic thinking (combination of simple parts into a complex whole) and attempts to represent place as a system of interrelated components, whose spatial configuration enables certain functions. This system view of place aims to bridge (practical) features of human understanding of place with rigid spatial representations. On the one hand, it maintains a connection between place and human reasoning by utilizing the supported functions to satisfy human purposes. On the other hand, the components that build the place facilitate the approximation of its spatial footprint, by taking advantage of their simpler spatial structure.

The proposed model of composition lies between the levels of functions and data. The former provides the necessary inputs to the level of composition, which feed the latter with thematic and spatial information that leads to an approximate spatial projection. On occasion, the sequence can also be reversed, i.e. it may follow the inductive route from components to the composition to function.

It is worth noting that a more detailed formalization of functions and components is beyond the scope of this paper. Consequently, we simplify the task here by assuming that descriptions of functions are derived from the text, in particular documents that provide widely acceptable definitions and descriptions of places such as dictionaries or encyclopedias. There are several works that use text as a source of information in place-related research (Khan, Vasardani, & Winter, 2013; Scheider & Purves, 2013; Vasardani, Winter, & Richter, 2013). However, since our methodology’s need for place-related knowledge goes beyond mere place names and related services, we require more elaborate sources, such as design standards and manuals (e.g. the retail design manual of the Department of Housing, Planning and Local Government in Ireland (https://www.housing.gov.ie/search/sub-topic/retail-planning)). Using these as a basis, we rely on specialists that can extract appropriately generalized rules that cover the maximum possible spatial configurations and compositions. Increasing automation by reducing reliance on specialists (e.g. as is the case of Alazzawi et al. (2012) for associating services to place types) is a matter of future work.

### 3.1. Composition and functionality of place

The level of composition associates the functions of a place with its physical structure. Individual compositions are viewed as instances of generic *design patterns* and describe the spatial configurations of the elements that enable a particular spatial function (or functions) characterizing a place. To formalize the level of composition, we propose the introduction of a rule set that translates every functional requirement into spatial structures on the ground. Conversely, by evaluating existing spatial structures by means of an appropriate rule set, one can detect whether or not a specific place is likely to support a particular set of functions.

The composition level is thus where the spatial structure of a place is generalized as a pattern, following the basic spatial requirements (such as elements, relations, and scale) of the corresponding function(s). These patterns (henceforth design patterns) are defined by the functions that a place offers and, upon decomposition, they approximate the minimum area where the place of interest is spatially projected. The composition of place is built upon components (see Section 3.2) and composition rules (see Section 3.3). The former refers to categories of physical entities, whereas the latter describes the associations that hold between the former.

Building the composition of a place is carried out through logical implications (see Section 3.4) for every function of the place under consideration. These constructs describe the required criteria that enable a function and are expressed as a combination of composition rules. Finally, the aggregated spatial projection of the components comprising the design pattern introduces the minimum area of confidence, within which the place under question can possibly be found. In the following subsections, we provide an extensive analysis of the building blocks of composition. Afterward, we list the proposed formalization of the design patterns of place as a rule-based synthesis, expressed in pseudo-code comprising first-order logic and set theory statements.

### 3.2. Components

People connect places to certain objects that trigger their interest, since these objects are necessary for the potential activities that an individual can realize within a place (Scheider & Janowicz, 2014, p. 102). With respect to the function-based model of place, the aforementioned objects refer to the constituents of a place, denoted as *components*. In particular, components represent concrete or abstract entities which enable, enhance, hinder, or block certain functions. The represented entities can be either particulars or universals. In the first case, a component represents a specific entity with particular characteristics, such as “runway R09”. On the contrary, in the case of universals, a component refers to a specific type of entities that share common characteristics or qualities such as “runway”. Each component is framed with properties that describe its thematic and geometric information, adhering to the following schema:

- **id** – a unique number used as reference for a component
- **type** – a category that describes the common features of a class of components
- **theme** – a list of thematic properties that semantically describe a component
- **geometry** – geometric properties that spatially describe a component

Thematic properties (theme) consist of name-value pairs and are used to semantically enrich a component; indicative such properties are: “the building was constructed in 1582” or “the road is a path and is walkable”. Geometric properties (geometry) also consist of name-value pairs and refer to the spatial information of a component; they are not dimension-dependent or limited to abstractions; therefore, any valid geometry can be used as property value. Consider the following examples expressed in Well Known Text (abbr. WKT) (http://www.opengeospatial.org/standards/wkt-crs):

- the building has geometry POLYGON ((30 10, 40 40, 20 40, 10 20, 30 10))
- the component has geometry MULTIPOINT (10 40, 40 30, 20 20, 30 10)
- the road has geometry LINESTRING EMPTY

Considering that components refer to both abstract or concrete entities, the assigned geometric properties are not limited by precision. Geometry may describe either fiat or bona fide objects (Smith, 2001), indicative components of an airport include detached entities such as terminals and runways, or vague areas such as safety and noise contour zones.

### 3.3. Composition rules

Components along with their semantics are essential for enabling or disabling particular functions: for example, an airport supports take-off and landing of passengers if and only if it is equipped with a runway and a terminal. However, the relations between the aforementioned components are equally important to determine the functioning of the airport: for instance, if the terminal is located within the runway, then the provision of both take-off and landing is no longer possible.

The relations that frame the components of a place are expressed with *composition rules*. They are defined as *n*-ary customizable predicates, where “*n*” stands for the degree of customization that an individual rule allows. For example, unary rules describe a single component, whereas binary rules refer to the association of two components. Design patterns include the following four composition rules: (a) occurrence, (b) correlation, (c) spatial relation, and (d) proximity. These formalize requirements about the existence and/or population on space, frequency of appearance, topology, and distance between components, respectively. Apart from the spatial configuration of components, a design pattern expresses the semantic properties as well, through the use of filters. Such filters are used as parameters for the composition rules. Three flavors of filters are supported: (a) property, (b) type, and (c) geometry.

We exemplify the expressiveness of the aforementioned rules using the design pattern that describes a civilian airport, considering the most common and expected functions found in the Wikipedia platform (https://en.wikipedia.org/wiki/Airport) and design regulations as they are determined by the Federal Aviation Administration of United States (https://www.faa.gov/airports/engineering/construction_standards/). We begin by defining appropriate filters. Property, type, and geometry filters specify the thematic and geometric properties that define a component, e.g. “the runway is made of asphalt”, “the terminal is a building”, “the taxiway falls within POLYGON ((30 10, 40 40, 20 40, 10 20, 30 10))”, respectively.

We continue with the composition rules. The occurrence is a unary rule that indicates the existence of a particular component; optionally, it can also include the minimum and/or the maximum number of occurrences, for instance, “there is one and only one component of type control tower”. The correlation rule is a binary association that expresses the frequency of appearance of two components, such as “the proportion of the number of aprons and terminals is less than one”, or, equivalently “an apron serves more than one terminal”. The topology between components is expressed with the binary rule of spatial relation. It is interpreted as spatial predicates derived from the DE-9IM model (Egenhofer & Franzosa, 1991), such as equals, contains, covers, disjoint, intersects, overlaps, crosses, and touches; for instance, “the terminal is disjoint with the runway”. Finally, the proximity rule expresses a binary distance relation, e.g. “the distance between the terminal and the parking facility is 500 m”. For the sake of simplicity, our formalization considers the latter as a quantitative function; however, qualitative values are also possible. For instance, the introduction of thresholds and nominal values can illustrate the vague concept of nearness (Herrlich, 1974). It is worth noting that the rules of spatial relation and proximity evaluate the given components in an exhaustive way. For instance, the spatial relation rule ”the terminals are disjoint with the runways” expresses that every terminal is disjoint with every runway. Similarly, the proximity rule “the distance between the terminals and the parking facilities is 500 m” states that every terminal is 500 m away from any parking facility.

### 3.4. Functional implication

The composition of place is finalized by associating each provided function with the corresponding composition rules, in the form of a logical statement, denoted as *functional implication*. This is defined as a logical implication inferring the enablement of a function out of a combination of particular components and rules. For instance, “the functions of takeoff and landing of aircraft are enabled if the following occurrence rule is evaluated as true: there must exist at least one component of type runway”. If a place is multifunctional, each function is deduced from a separate functional implication. In addition, more complex functions can be deduced from simpler ones. For example, “the function of aircraft taxiing is enabled if the following hold:

1. the occurrence rule, which describes the existence of a component of type taxiway
2. the function of housing aircraft is enabled (due to the existence of an hangar)
3. the function of repair aircraft is enabled (due to the existence of an apron)
4. the function of take off and landing of aircraft is enabled (due to the existence of a runway)
5. the spatial relation rule, which states that the spatial association of the component taxiway with each one of runway, hangar, and apron, is touch”

### 3.5. Formalization

In the following, we assume that STR is a countable, nonempty set of alphanumeric values.

**Definition 3.1**. (**Component**) Let GEO denote a nonempty set of valid geometries, which are vector geometries with types able to be represented by the distinct geometric objects of WKT. Also, let ATR stand for a set of name-value pairs of the form $\left\langle p\_name,p\_value\right\rangle$, with p_name,p_value∈STR, representing properties. A component is defined as a physical entity described by the following schema: Component(id, type, geom, ATR), where id,type∈STR, representing the identifier and type of the component, respectively, and geom∈GEO representing its geometry.

**Definition 3.2**. (**Auxiliary Methods for Components**) Let CMP denote the set of all possible components in the current composition, conforming to the schema stated in Definition 3.1, and $c,c_{1},c_{2}\in CMP$. Also, let SR denote the set of all DE-9IM spatial relations. The following auxiliary methods are available:

- id(c):CMP→STR, returns the id of component c.
- type(c):CMP→STR, returns the type of component c.
- geom(c):CMP→GEO, returns the geometry of component c.

○ geomT(geo):GEO→STR, a complementary method that returns the geometric type of the geometry geo, based on the geometric objects defined in WKT.

•property(c,p_name):CMP×STR→STR, returns the value of property p_name for component c.
•$s\_r(c_{1},c_{2}):CMP\times CMP\to SR$, returns the spatial relation between components $c_{1}$ and $c_{2}$.
•$dst(c_{1},c_{2}):CMP\times CMP\to R^{+}\cup^{\left\{0\right\}}$, returns the geometric distance between components $c_{1}$ and $c_{2}$.

**Definition 3.3**. (**Composition Filtering**) Let CMP denote the set of all possible components in the current composition, defined according to Definition 3.1. The following methods apply filters on CMP in order to define sets FIL⊆CMP, which conform to specific characteristics:

- PropertyFilter(p_name,p_value):STR×STR→FIL, with c∈FIL iff property(c,name)=p_value, selects all the components that have a property named p_name with assigned value p_value
- TypeFilter(tp):STR→FIL, with c∈FIL iff type(c)=tp, selects all the components that are of type tp
- GeomFilter(sr,geometry):SR×GEO→FIL, with c∈FIL iff s_r(geom(c),geometry)=sr, selects all the components that are spatially associated with the geometry via the spatial relation sr
- GeomTFilter(geometry_type):STR→FIL, with c∈FIL iff geomT(geom(c))=geometry_type, selects all the components that have the given geometry_type

**Definition 3.4**. (**Composition Rules**) A *composition rule* is a logical predicate that can be evaluated to true or false, depending on the values of its attributes. The following composition rules are defined:

- Occurrence(A,N), with A ⊆CMP, $N\subseteq N\cup^{\left\{0\right\}}$, carrying the semantics that $\left|A\right|\in N$: the count of the given set of components A must be a number that belongs to the non-negative numerical set N.
- Correlation(A,B,N), with A,B⊆CMP, $N\subseteq R^{+}$, carrying the semantics that $A\neq \emptyset \wedge B\neq \emptyset \wedge \left|A\right|/\left|B\right|\in N$: the ratio between the counts of the sets of components A and B must be a positive non-zero number that belongs to the numeric set N.
- SpatialRelation(A,B,R), with A,B⊆CMP, R⊆SR, carrying the semantics that A≠∅∧B≠∅∧R≠∅∧∀a,b(a∈A∧b∈B→s_r(a,b)∈R): the spatial relation that holds between any combination of components from the sets A and B must belong to the set R.
- Proximity(A,B,N), with A,B⊆CMP, $N\subseteq N\cup^{\left\{0\right\}}$, carrying the semantics that A≠∅∧B≠∅∧∀a,b(a∈A∧b∈B→dst(a,b)∈N): the distance between any combination of components from the sets A and B must belong to the non negative numeric set N.

**Definition 3.5**. (**Functional Implication**) A *function* is formalized as a logical predicate with one or more components as attributes. Let F denote the set of all functions in the current composition and CR denote the composition rules in Definition 3.4. A *functional implication* is defined as a logical implication of the form f←ϕ, with f∈F and ϕ a (first-order) logical formula, formed by logical connectives ¬, ∧and ∨, composition rules in CR and possibly other functions in F. The semantics is that the function in the head of the implication is supported if the logical formula in the body holds.

For instance, let the function “takeoff” be enabled in a place, if the latter is equipped with at least one runway of length at least 500 m. The functional implication that describes this example is formulated as follows: ∩PropertyFilter(width,500),[1,∞)). To evaluate the rule, three steps are required: (a) from the available components select those that are of type *runway* and those whose length is at least 500 m; (b) calculate the intersection on the extracted subsets of components, resulting in a new set of components which contains all the runways whose length is at least 500 m; (c) apply the composition rule of occurrence on the new set – the “takeoff” function is enabled only if the set is not empty, which means that there is at least one component that has the features mentioned above.

## 4. Demonstration

In this section, we demonstrate the applicability of the proposed model using the example of place search. We focus on the identification of places characterized by particular functionality, specifically the shopping areas in the city of London. The experiment is divided into two phases: (a) spatial composition of place and (b) assignment of functions on space. The first phase aims to construct a design pattern that describes a place that provides the predefined functions of a shopping area. The second phase focuses on locating all the spatial regions identified by the aforementioned design pattern. Considering that “shopping area” can be a vague term, we focus here on complexes designed to offer similar functionality to shopping malls, which are the most recognizable examples of shopping areas in western countries. Our search query thus becomes: “Locate all the shopping areas, in other words, locate all the places that operate as a shopping mall in Greater London”. In order to avoid confusion, from this point onward, any reference to places that operate similarly to a shopping mall will be denoted as shopping area.

### 4.1. Assumptions and thematic prerequisites

The functioning of a shopping area, in our example, is determined by analyzing the possible human activities or interactions supported by a shopping mall. Shopping malls provide a number of functions that are the result of existing knowledge and evolution of Western culture. In particular, these functions portray how the majority of western civilizations would expect a shopping mall to operate. Information about these entities is usually documented in nonscientific narratives that aim to illustrate widely acceptable definitions, i.e. Oxford Dictionary (https://en.oxforddictionaries.com/definition/mall) and Wikipedia (https://en.wikipedia.org/wiki/Shopping_mall).

For the sake of simplicity, we assume a basic version of shopping mall that includes the following functions: “shopping opportunities” (denoted as $F_{SO}$), “basic shopping facilities” ($F_{BS}$), “special shopping facilities” ($F_{SS}$), “sustenance services” ($F_{S}$), “entertainment services” ($F_{E}$) and “luxury services” ($F_{L}$). Additional functions may be “trading products outside shops” ($F_{TP}$), “resupply of shops” ($F_{R}$), “walkability within an area” ($F_{W}$), “access for drivers” ($F_{AD}$), “access for non-drivers” ($F_{AN}$), “compatible products” ($F_{C}$), and “safety from vehicles” ($F_{VS}$). A place supporting the aforementioned functions allows customers to explore a variety of shops and amenities, ranging from basic to more advanced needs, while excluding incompatible goods such as constructive material, bulky products, and so on. All of the aforementioned services are provided within a secure, walkable, and accessible environment. In addition, it facilitates access to high-capacity roads and provides services for drivers and nondrivers. The rules that frame a shopping area can be inferred from narratives or plans that describe the designing and architectural trends of shopping malls such as Wikipedia entries (https://en.wikipedia.org/wiki/Shopping_mall), reports from the American Planning Association (https://www.planning.org/pas/reports/report59.htm), or guides for urban infrastructure (https://www.tccs.act.gov.au/__data/assets/pdf_file/0005/396878/ds17_shoppingcentres.pdf).

Several assumptions are made in order to develop the current example based on the standards of the western type shopping malls. In particular, the spatial composition focuses on shopping areas compatible with Western culture standards. For instance, basic shopping includes clothing and household products, whereas advanced shopping includes special and more luxury-oriented needs that aim to improve the quality of life, such as sports, health, and beauty products. Similarly, the supported amenities are divided into three categories: (a) food and drink, (b) entertainment, and (c) business-related needs. Furthermore, we assume that walkability is supported by ensuring that distance among shops, amenities, parking areas, service roads, and bus stations does not exceed 5 min of average walking speed (∼5 km/h), which corresponds approximately to 400 m, as it is estimated by a survey on the distance in human transit (https://humantransit.org/2011/04/basics-walking-distance-to-transit.html). Also, the distance between shops and highways is assumed to be under 5 km, which corresponds to 5 min of average driving speed (∼64 km/h).

**Figure 1.**
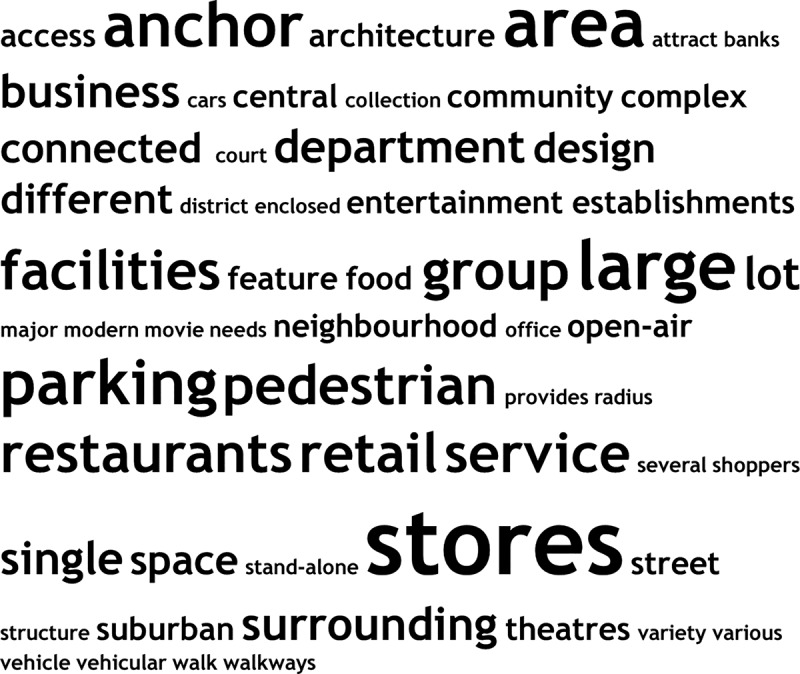
Word cloud based on the term “Shopping mall” using various text sources.

The aforementioned assumptions are derived from the definitions of a shopping mall (and relevant terms) as provided by various sources: Oxford Dictionary (oxforddictionaries.com), Merriam–Webster (merriam-webster.com), Collins Dictionary (collinsdictionary.com), Cambridge Dictionary (dictionary.cambridge.org), Dictionary.com (dictionary.com), Business Dictionary (businessdictionary.com), Encyclopedia.com (encyclopedia.com), Encyclopedia Britannica (britannica.com), and Wikipedia (wikipedia.org). This claim can be justified by observing the corresponding word cloud depicted in Figure 1. Note that these assumptions underlie the composition of a particular type of shopping area and should not be considered exhaustive or universal. However, given the generalized formalization of the composition model of place, it is possible to describe different forms of shopping areas based on alternative assumptions that reflect different kinds of urban areas or cultures. Providing such alternative assumptions is beyond the scope of the current paper.

### 4.2. Spatial composition of place

The process of spatial design is initiated with the aforementioned functions. Following the guidelines gained from narratives, we conclude to the main categories of components that will be used for the current example:

- **Shop**: (0, Shop, GEOMETRY EMPTY, [goods = *])
- **Amenity**: (1, Amenity, GEOMETRY EMPTY, [service = *])
- **Road**: (2, Highway, LINESTRING EMPTY, [pedestrians = *])
- **Open area**: (3, Surface, POLYGON EMPTY, [walkable = *])

The * symbol is used to denote an unbounded set of possible values. Table 1 illustrates the essential components of shopping areas. Note that the walkability property demands the individual component to be a solid, flat surface on which a person can walk. Then, we introduce the design pattern depicted in Table 2. The latter indicates that a candidate shopping area must offer certain shopping and leisure services, which are supported by the existence and spatial organization of particular components. Some indicative examples are provided and interpreted as follows. A candidate shopping region supports the function of walkability $F_{W}$ if there is a walkable surface that includes at least two facilities (shops or amenities), whose distance does not exceed 400 m; in addition, this area must be intersected by service roads. A region offers shopping opportunities ($F_{SO}$), if it supports the walkability function and there are at least two anchor shops or five basic shops that fall within its surface. The rest of the functional implications are interpreted in a similar way.

**Table 1.** Set of components of a shopping area.

Variable	Component	Filter
$C_{S}$	Shop	Type_Filter(Shop)
$C_{A}$	Amenity	Filter(Amenity)
$C_{F}$	Facilities	$C_{S}\cup^{C_{A}}$
$C_{W}$	Walkway	${\%}_{F}ilter(Surface)\cap$Prop_Filter(walkable,true)
$C_{OW}$	Outer Walkway	$Type\_Filter{(}^{\prime \prime }Surface^{\prime \prime })\cap$$Prop\_Filter{(}^{\prime \prime }walkable^{\prime \prime }{,}^{\prime \prime }true^{\prime \prime })$
$C_{H}$	Motorway	$Type\_Filter{(}^{\prime \prime }Road^{\prime \prime })\cap$$Prop\_Filter{(}^{\prime \prime }pedestrians^{\prime \prime }{,}^{\prime \prime }false^{\prime \prime })$
$C_{Sr}$	Service Road	$C_{H}\cap \;Prop\_Filter{(}^{\prime \prime }pedestrians^{\prime \prime }{,}^{\prime \prime }true^{\prime \prime })$
$C_{P}$	Parking place	$C_{A}\cap \;Prop\_Filter{(}^{\prime \prime }service^{\prime \prime }{,}^{\prime \prime }parking^{\prime \prime })$
$C_{B}$	Bus station	$C_{A}\cap \;Prop\_Filter{(}^{\prime \prime }service^{\prime \prime }{,}^{\prime \prime }transportation^{\prime \prime })$
$C_{An}$	Anchor Store	$C_{S}\cap^{P}rop\_Filter{(}^{\prime \prime }goods^{\prime \prime }{,}^{\prime \prime }multipurpose^{\prime \prime })$
$C_{Sb}$	Basic Shop	$C_{S}\cap^{P}rop\_Filter{(}^{\prime \prime }goods^{\prime \prime }{,}^{\prime \prime }basic^{\prime \prime })$
$C_{Se}$	Special Shop	$C_{S}\cap^{P}rop\_Filter{(}^{\prime \prime }goods^{\prime \prime }{,}^{\prime \prime }special^{\prime \prime })$
$C_{Su}$	Uncommon Shop	$C_{S}\cap^{P}rop\_Filter{(}^{\prime \prime }goods^{\prime \prime }{,}^{\prime \prime }uncommon^{\prime \prime })$
$C_{As}$	Food court	$C_{A}\cap^{P}rop\_Filter{(}^{\prime \prime }service^{\prime \prime }{,}^{\prime \prime }sustenance^{\prime \prime })$
$C_{Ae}$	Entertainment	$C_{A}\cap^{P}rop\_Filter{(}^{\prime \prime }service^{\prime \prime }{,}^{\prime \prime }entertainment^{\prime \prime })$
$C_{Al}$	Luxury services	$C_{A}\cap^{P}rop\_Filter{(}^{\prime \prime }service^{\prime \prime }{,}^{\prime \prime }luxuryneeds^{\prime \prime })$

**Table 2.** Design pattern of a shopping area.

CMP:$\left\{C_{F},\;C_{S},\;C_{A},\;C_{H},\;C_{Sr},\;C_{W},\;C_{OW},\;C_{B},\;C_{P},\;C_{An},\;C_{Sb},\;C_{Se},\;C_{Su},\;C_{As},\;C_{Ae},\;C_{Al}\right\}$	
Functional Implications	
Functions (F)	Logical Formula
$F_{W}(C_{F},C_{W},C_{Sr})$	$\left(Occurrence(C_{F},[2,\infty ])\wedge Proximity(C_{F},C_{F},(0,400m]))\wedge (Occurrence(C_{W},[1,1])\wedge \;S\_Relation(C_{W},C_{F},[contains])\wedge Occurrence(C_{Sr},N)\wedge S\_Relation(C_{Sr},C_{W},[intersects])\right)$
$F_{SO}(C_{An},\;C_{S})$	$F_{W}\wedge (Occurrence(C_{Sb},[5,\infty )\wedge S\_Relation(C_{W},C_{Sb},[contains]))\vee (Occurrence(C_{An},[2,\infty )\wedge S\_Relation(C_{W},C_{An},[contains]))$
$F_{BS}(C_{Sb})$	$F_{SO}\wedge Occurrence(C_{Sb},N)$
$F_{SS}(C_{Se})$	$F_{SO}\wedge Occurrence(C_{Se},N)$
$F_{C}(C_{Su})$	$F_{SO}\wedge Occurrence(C_{Su},N)\to Correlation(C_{S},C_{Su},[1,\infty ))$
$F_{TP}(C_{S},C_{A})$	$F_{W}\wedge Occurrence(C_{A},N)\to Correlation(C_{S},C_{A},[2,\infty ))$
$F_{S}(C_{As})$	$F_{TP}\wedge Occurrence(C_{As},N)$
$F_{E}(C_{Ae})$	$F_{TP}\wedge Occurrence(C_{Ae},N)$
$F_{L}(C_{Al})$	$F_{TP}\wedge Occurrence(C_{Al},N)$
$F_{R}(C_{W},C_{H})$	$F_{SO}\wedge (Occurrence(C_{H},N)\wedge Proximity(C_{W},C_{H},(0,5000m]))$
$F_{AD}(C_{OW},C_{W},C_{P})$	$F_{W}\wedge Occurrence(C_{OW},[1,1])\wedge S\_Relation(C_{OW},C_{W},[contains]))\wedge (Occurrence(C_{P},N)\wedge S\_Relation(C_{OW},C_{P},[contains]))$
$F_{AN}(C_{OW},C_{W},C_{B})$	$F_{W}\wedge Occurrence(C_{OW},[1,1])\wedge S\_Relation(C_{OW},C_{W},[contains]))\wedge (Occurrence(C_{B},N)\wedge S\_Relation(C_{OW},C_{B},[contains]))$
$F_{VS}(C_{H})$	$F_{W}\wedge Occurrence(C_{H},N)\to S\_Relation(C_{W},C_{H},[disjoint]))$

### 4.3. Assignment of functions on space

Applying the proposed design pattern using actual data allows the localization of the shopping areas that may exist in a specific region. The selected area of study is Greater London, and the spatial features of interest are extracted from freely available information provided by OpenStreetMap (https://www.openstreetmap.org/) and GeoNames (http://www.geonames.org/). In particular, all the registered points and lines that fall within the area of study were acquired and classified based on the data organization guidelines provided in the OpenStreetMap portal (https://wiki.openstreetmap.org/wiki/Category:Keys). A summary of the spatial data that were used in this example is provided in Table 3, and an indicative overview of the classification is shown in Table 4. It is worth noting that the spatial analyses that are presented in the rest of this section are regarded as demonstrative solutions and do not suggest efficient or effective techniques of processing.

**Table 3.** Data used in the demonstration.

Spatial Reference System	EPSG:32,630-WGS 84/UTM zone 30N (Projected)
Bounding Box for London	672,384.00,5,685,190.00: 732,284.00,5,731,440.00
# of Points of Interest	284,315
Schema for Points	fid (Integer), osm_id (String), name (String), barrier (String), highway (String), ref (String), address (String), is_in (String), place (String), man_made (String), other_tags (String)
# of Lines	63,723
Schema for Lines	fid (Integer), osm_id (String), name (String), highway (String), waterway (String), aerialway (String), barrier (String), man_made (String), z_order (Integer), other_tags (String)

**Table 4.** Types of spatial features according to openStreetMap portal.

Shop		Amenity		
*Basic*	*Special*	*Sustenance*	*Entertainment*	*Business*
General Department Store, Food & Beverages, Clothing-Shoes & Accessories	Health & Beauty, Electronics, Outdoors Sports & Hobbies	Pub, Cafè, Restaurant, Food Court, Fast Food	Cinema, Arts, Playground	Bank, ATM, Healthcare
**Transportation**		**Road**		
Car & Bicycle Parking, Bus station, Taxi		Service road, Highway, Highway junction		

**Figure 2.**
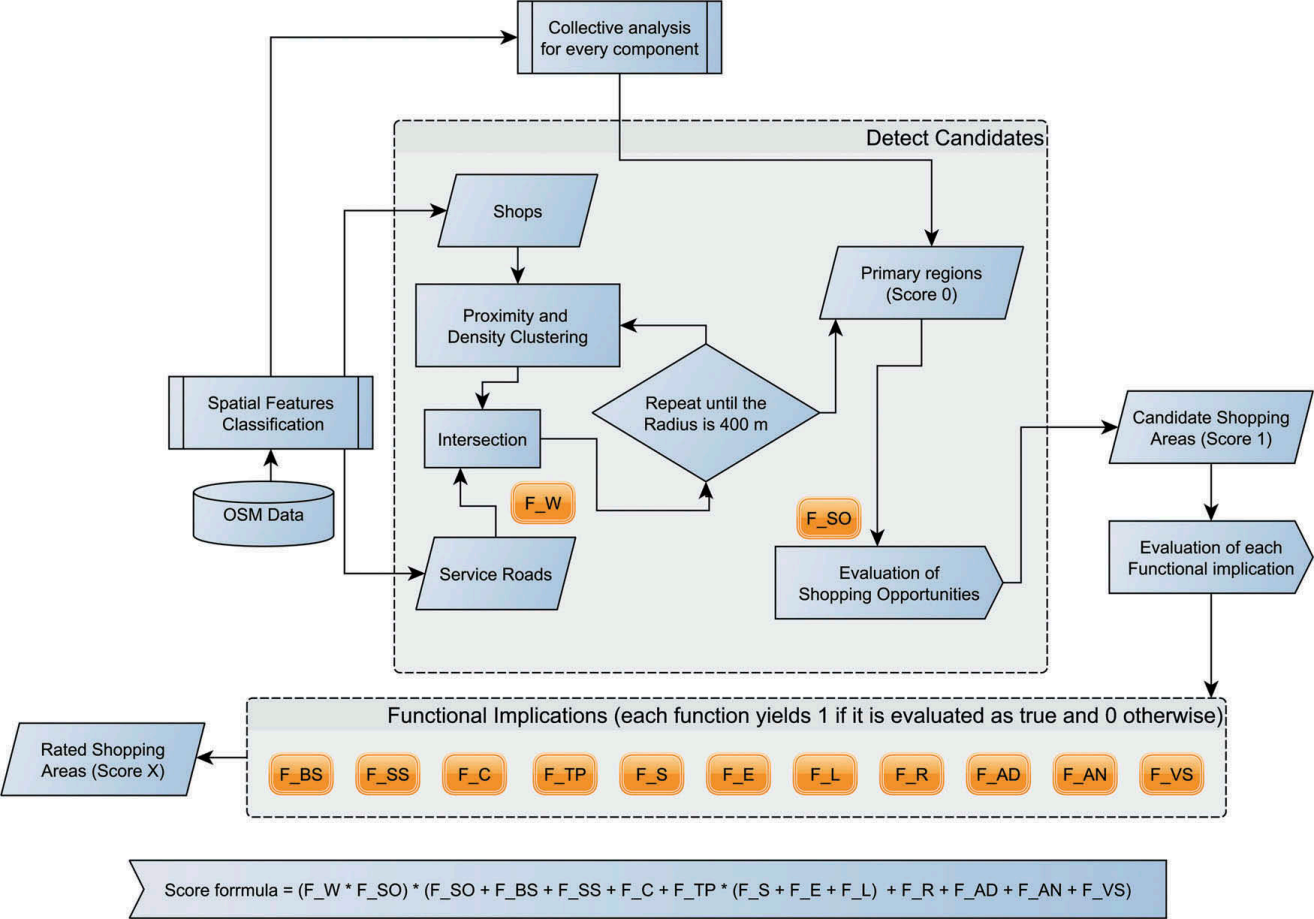
Process used to assign functions and to attribute scores.

The process of assigning functions on space is illustrated in Figure 2 and consists of two phases: (a) the detection of the regions that qualify as candidate shopping areas and (b) the evaluation of functional possibilities for each candidate area. The number of functional requirements that a candidate satisfies indicates the confidence of this area to operate as an advanced shopping mall. The detection process is initiated by checking if the function $F_{W}$ is satisfied, based on the corresponding functional implication in Table 2. Areas that fulfill the latter are designated as primary regions and are subjected to a collective analysis. In particular, in this step, we aggregate and calculate the occurrence of different types of components within or the surrounding areas of the primary regions. Then, a region is converted into a candidate shopping area if the function $F_{SO}$ holds.

The process of discovering functional possibilities is achieved by employing a rule-based algorithm that rates each candidate according to the successful evaluation of the remaining functions. A formula used for the score is depicted in Figure 2 and is explained here. Every candidate starts with the minimum rating, equal to 1. Then, for each candidate, we examine whether its spatial configuration allows different functions to be enabled. The rating of a candidate is increased by 1 for each successful evaluation. The highest rating, equal to 10, is granted when a shopping area represents an advanced place that operates as a fully functional shopping mall, with respect to our assumptions listed in Section 4.1.

## 5. Results and discussion

The objective of the demonstration listed in Section 4 is the identification and localization of every place in Greater London that is likely to operate as a shopping mall. In addition, the candidate shopping areas are attributed to ratings that quantify the functionality of each subject, in terms of the number of provided functions. We describe and evaluate the results of this place-based query in two phases: first by analyzing the overview of the results and then focusing on a small central subregion in the city, in order to provide finer details and better visualization.

**Figure 3.**
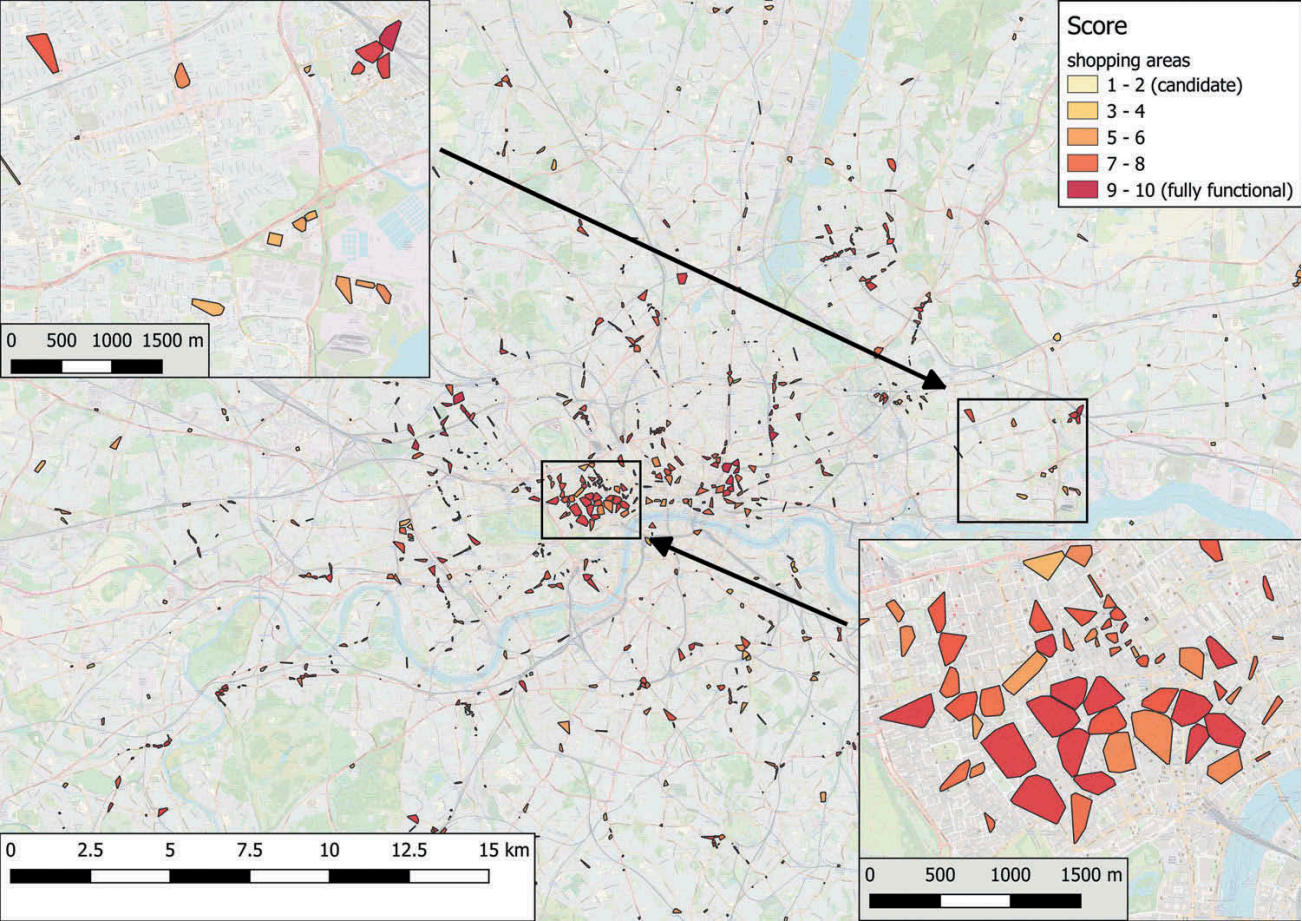
Identification and rating of shopping areas in Greater London.

The algorithm identified 2408 primary regions, of which 1206 are recognized as candidate shopping areas. Figure 3 depicts an overview of the results of the place search query. The majority of the shopping areas is concentrated on the center of the city, creating a “hot” core of highly rated shopping areas, which indicates the busy areas such as strip malls or business districts. The shopping areas outside the city center are more sparse, forming small “veins” that spread throughout the city, and they are more likely to have a lower rating than the ones inside. This result indicates the development of the city through time as people tend to move out of the city center, and consequently their needs drive the improvement of surrounding areas.

**Figure 4.**
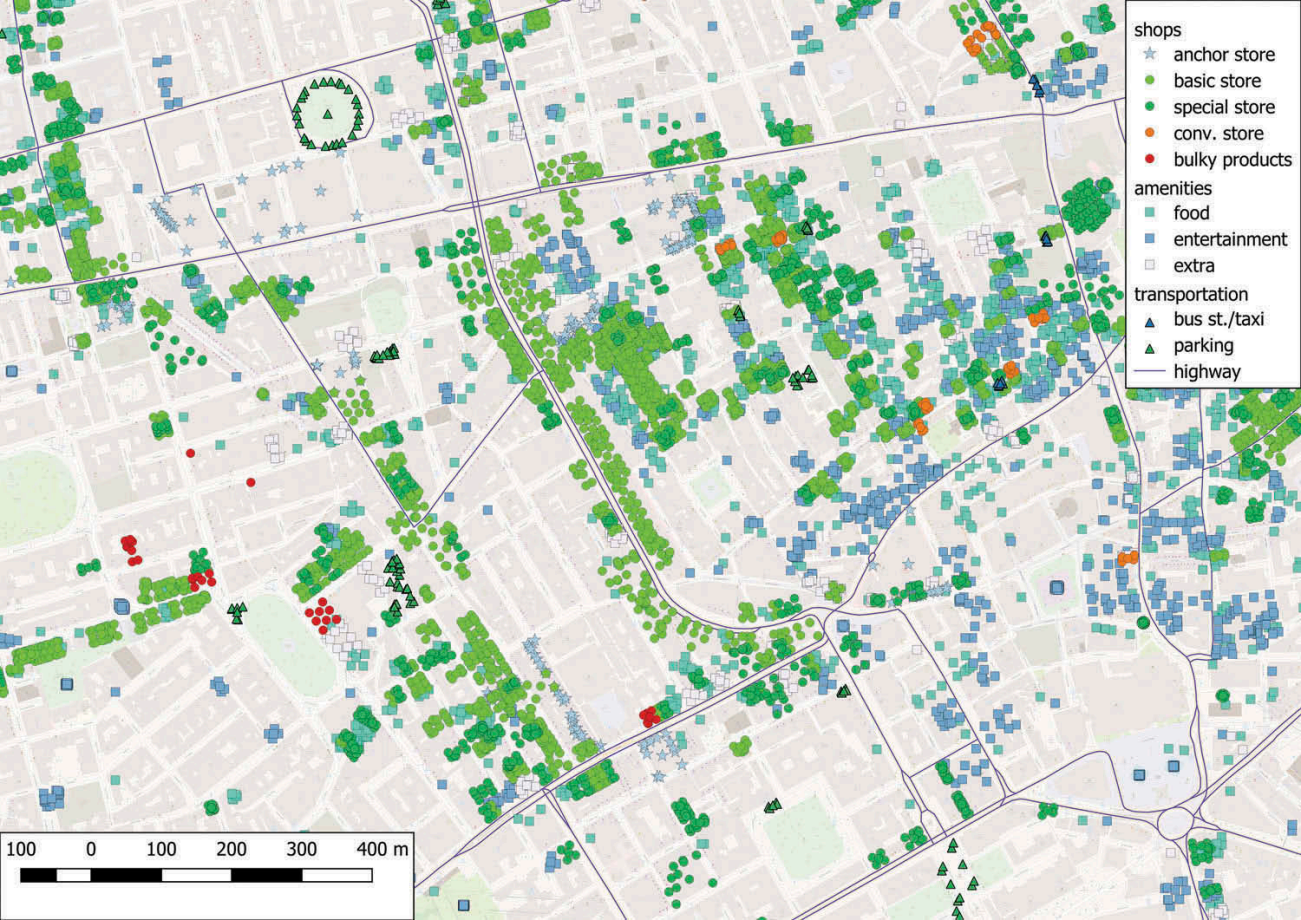
Spatial features in a central subregion of Greater London.

**Figure 5.**
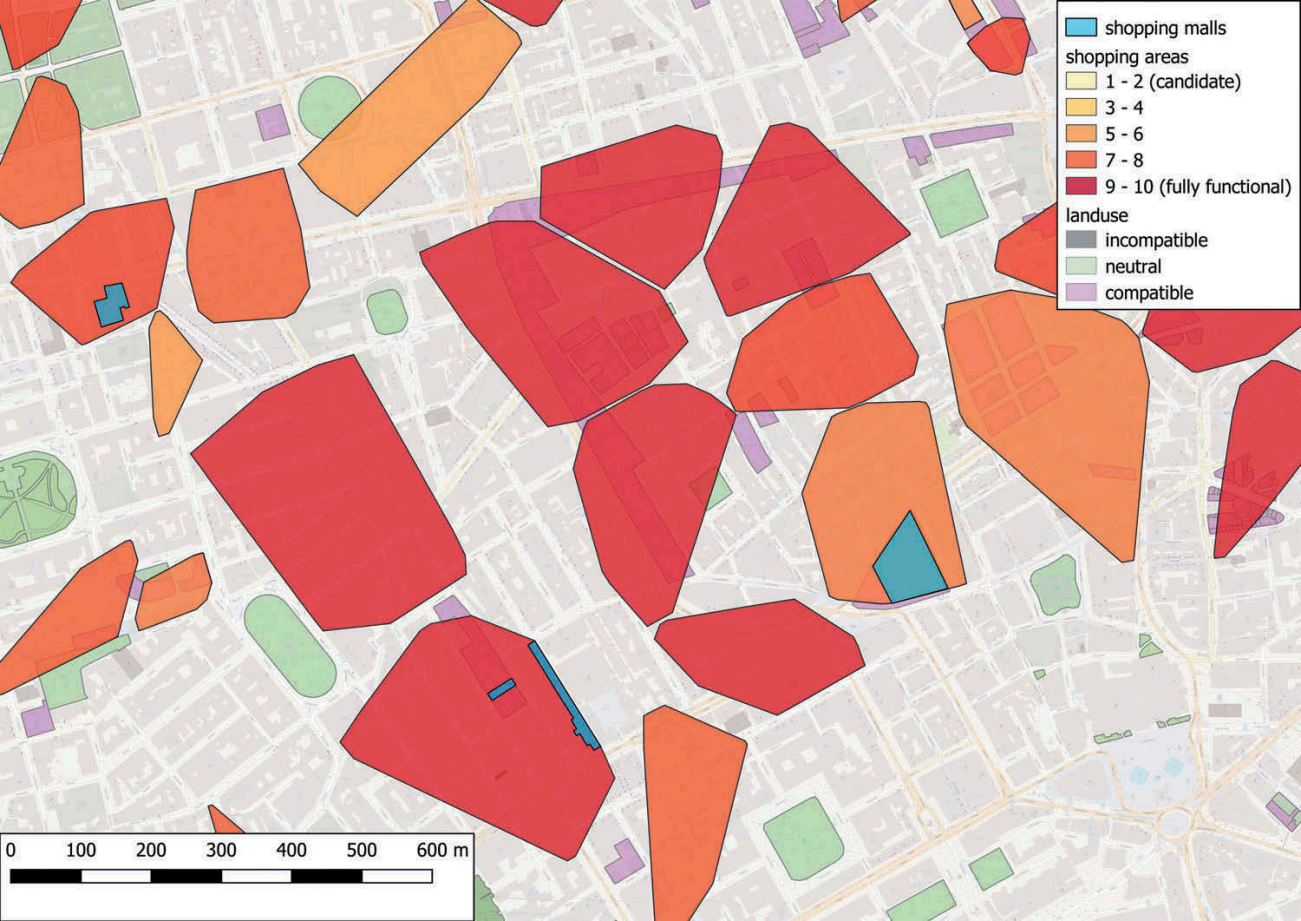
Shopping areas in a central subregion of Greater London.

Focusing on a small central subregion, Figure 4 shows the arrangement of the raw spatial data used for this example, whereas Figure 5 illustrates the detected and classified shopping candidates that conform to the proposed design pattern 2. Comparing Figures 4 and 5, it is concluded that the highest-rated shopping areas refer to walkable regions characterized by a dense concentration of various shops and amenities, that are surrounded by multiple transportation hubs. On the contrary, areas with a high population of amenities are attributed to lower ratings, since they are specialized in trading services instead of products. The same applies in cases of shopping areas that offer a limited variety of options or areas that have an increased number of uncommon facilities, such as convenient stores or stores dedicated to sell bulky products (e.g. motor vehicles or car repairs). It is worth noting that the proposed design pattern, and hence the detection process, relies on the quality of the provided facilities instead of the quantity of corresponding components. Specifically, an increased number of basic shops in an area will not yield a higher rating for this area; instead, it is simply interpreted as providing the basic shopping function $F_{Sb}$.

To evaluate the results, we use third-party sources, specifically Geonames and Open Street Map land use classification. A region is considered to be a valid result if it operates as a shopping area; this depends on one of the following criteria being true: (a) collective analysis suggests that the region is widely acceptable to be a shopping area and (b) the region does not overlap with areas that contain exclusive components. For the first criterion, we rely on the list of regions labeled as shopping malls in Geonames, based on user-contributed data. The second criterion is based on the definition of logical possibility: a fact is possible if there is no knowledge otherwise. With respect to our model, a place is possible to operate as a shopping area if there are no components that make this operation impossible, such as industrial units within a candidate area. We assume that a region is incompatible, neutral, or compatible to host a shopping area if it falls within a land use unit included in Table 5.

**Table 5.** Land use types and compatibility with a shopping area.

Land Use Type	Status
Forest, farm, scrub, industrial, quarry, meadow, cemetery,	Incompatible
Allotments, military, nature reserve	
Grass, recreation ground, orchard, residential, park, heath	Neutral
Retail, commercial	Compatible

**Figure 6.**
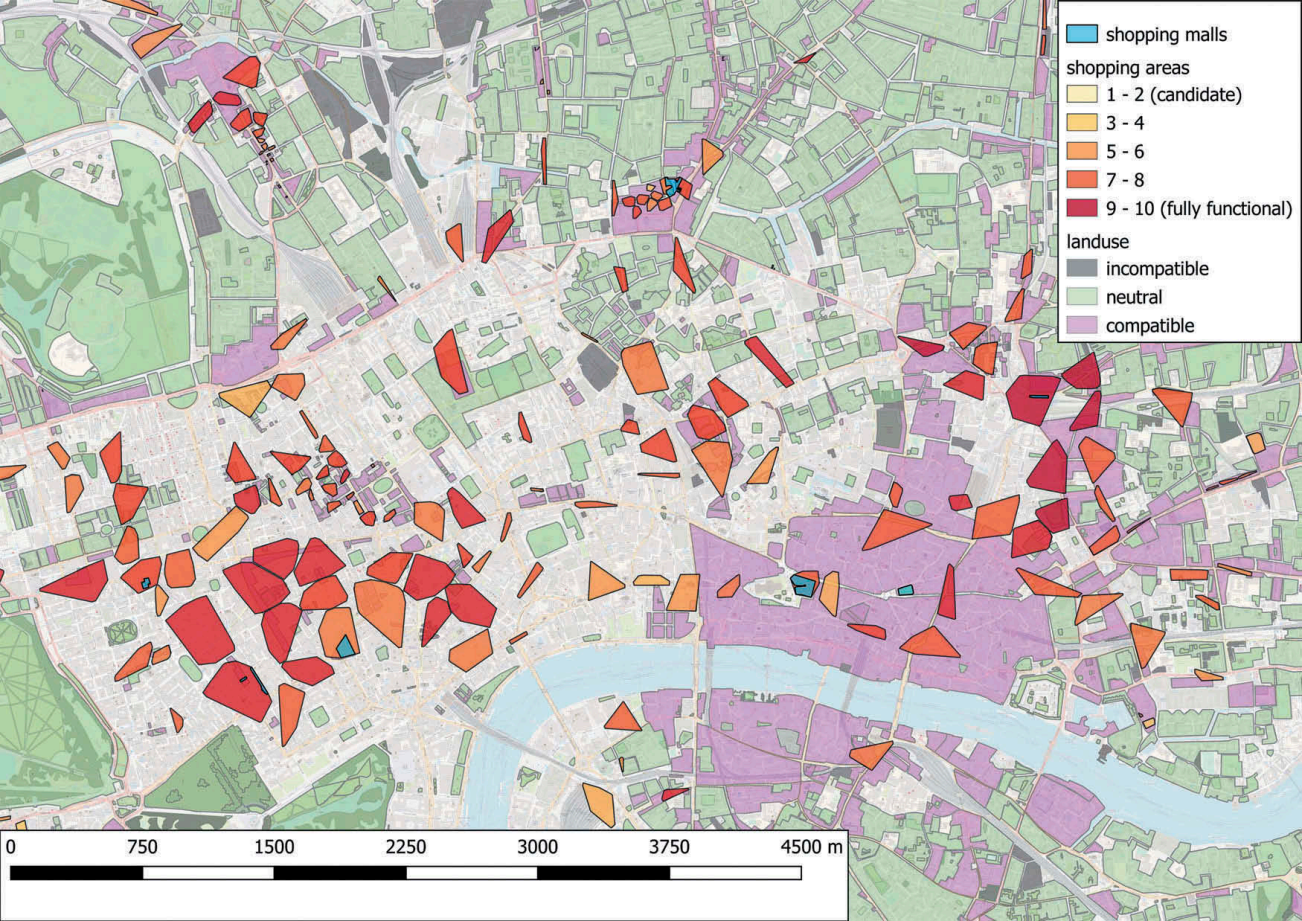
Evaluation of the validity of shopping areas using land use compatibility.

Figure 6 depicts a subregion of the selected area of study and shows the overlay between land use units and shopping areas. Every area labeled as shopping mall according to GeoNames (shown as circles) corresponds to one or more shopping areas calculated by our framework. This result verifies the completeness of our approach. Apart from these results, there are additional ones, for which we need to confirm that they can operate as shopping areas, according to the second criterion above. The results of this process are shown in Table 6, where 99.99% of the identified shopping areas do not include components that hinder the desired functionality.

**Table 6.** Evaluation of the shopping areas included in results.

**Malls (65)**	Count	Percentage
Contained in shopping areas	37	57%
Intersecting with shopping areas	65	100%
**Shopping areas (1206)**		
Contained in incompatible land use units	9	0.007%

**Figure 7.**
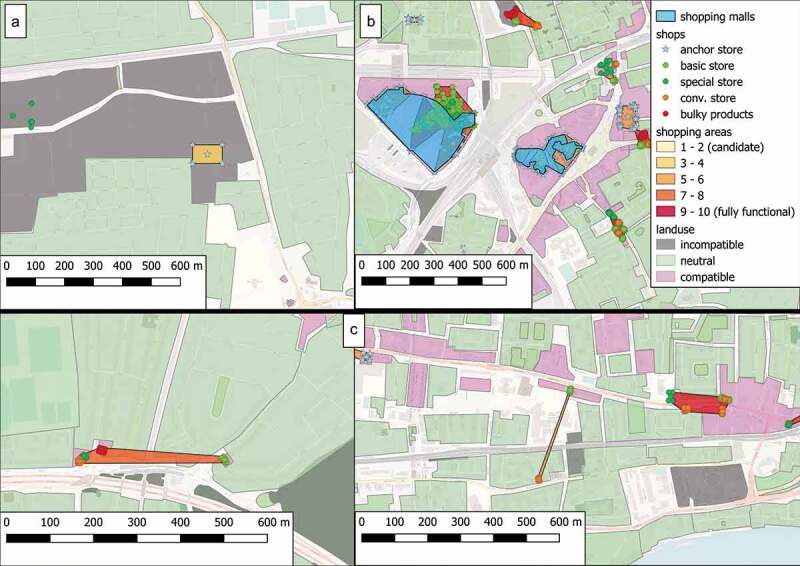
Common errors during the process of identification and localization of shopping areas.

Figure 7 indicates the three common errors that our identification method encounters: (a) discover a shopping area within an incompatible zone, (b) over-segment the spatial extent of a shopping mall, and (c) calculate a distorted spatial extent for a candidate area. The first case refers to outliers while evaluating the second criterion (mentioned above); a possible reason for this may be insufficient data. The second case occurs when a real shopping mall violates the walkability rule that we assumed in Section 4. Finally, the problem of the distorted shopping areas is a side effect of the clustering methods that we employed in our demonstration.

### 5.1. Advantages

The aforementioned areas indicate places that are highly rated in terms of their shopping-related functionality, in spite of not being labeled as an actual shopping mall. These areas exemplify the capabilities of the composite view of place. In the proposed framework, the shopping areas are not bound to text-based descriptions, semantics, or designation methods, as is common in gazetteer-related approaches; instead, places are treated as a system whose identity and location depends on the spatial organization of the included components and hence, the provided functions. Another important advantage of the proposed formalization is dynamic adjustment. In particular, the design patterns can be easily modified to answer more detailed place search queries. An example of such a query is: “locate every place that operates as a shopping area, offers sustenance facilities and the average network distance of two facilities is 200 m”. Such detailed queries are not answerable by traditional place-based techniques, such as gazetteers.

A notable feature of the composite view of place is the approximation of its spatial extent. Particularly, the resulted shopping areas are depicted as crisp regions, whose boundaries express the minimum area within which the desired functions are enabled. To the best of our knowledge, this has not been attempted before, since the traditional spatial projection of place is limited to points of interest or arbitrary geometries.

With respect to similar approaches of describing place which are driven by the essential kinds of place inference (Tuan, 1977), such as the work of Scheider and Janowicz (2014), the proposed approach is comparatively more objective, since it does not depend solely on an individual’s capabilities or judgment. Instead, it relies on widely accepted definitions of places and functions that resemble Alexander’s property of *echo*(Vasardani, Tomko, & Winter, 2016). Furthermore, our approach benefits from relational semantics and spatial organization among components that frame a place, instead of looking at it as a bag of objects.

Our approach aspires to occupy the middle point between works that focus solely on knowledge representation, such as ontologies and Scheider and Purves (2013), and purely data-driven methods (e.g. Gao et al. (2017)). In this way, several practical facets of place can be captured, while maintaining a sufficient spatial projection. Moreover, the proposed formalization and associated function-based search methodology have been fully implemented, allowing them to be easily integrated into traditional GIS, imbuing the latter with place-related capabilities. Additionally, since results are based on the function-based model of place and not merely a collection of data, it is straightforward to explain, based on the model, why one region is considered to function as a particular place, while another is not.

### 5.2. Limitations

Our approach relies on the assumption that place offers particular functions, which should be clearly defined and widely agreed. In addition, these functions must be associated with construction trends or standards, which in turn will facilitate the successful extraction of the required components and their in-between associations. If such knowledge is unavailable, e.g. because relevant narratives or other sources cannot be procured, then design patterns cannot be determined or formalized as defined in this work. In such cases, the need for alternative sources is raised and can be achieved by consulting specialists (i.e. architects), conducting surveys, or applying data-driven approaches. The latter inspires an interesting direction, in which the theoretical knowledge is potentially substituted or augmented with empirical data.

Also, different cultures may define similar places in a different manner: the expected functions of a shopping area in the Eastern world may include the trade of animals which is uncommon in the Western world. The same holds for differences in functionality provided by a place in different times of a day, week, or year: for instance, a football field may also host non-football events, such as concerts, during some days of the year. We aim to address these dependencies of place in future work, by allowing place types to be described by multiple design patterns.

It should be noted that the described methodology is affected by the inability to indicate ground truth: in our example, it is straightforward to say where a shopping area is located, but to state that a particular area is not shopping-related we can only rely on aggregations such as land use. Hence, a more accurate evaluation of the presented results could be possible through survey-based processes where people interested in a particular place functionality comment on whether higher-scored areas better serve their purposes.

Finally, the adopted function-based view of place does not allow the representation of fundamental elements that people associate with places, such as sentiments, sense of place, purpose of existence, and others. We acknowledge that these facets require more elaborate tools that are not confined by logic representations. Furthermore, treating the spatial extent of places using crisp boundaries gives an estimation of where a place ends. However, places can also be heterogeneous areas or spatially fragmented entities.

## 6. Conclusions

This paper contributes to the formalization of place utilizing a composite approach of formalizing place that builds upon the function-based model of place. In particular, we proposed a detailed model of composition that regards place as a system of interrelated components that enable or disable a particular functionality. The model lies between works that focus solely on knowledge representation, such as ontologies, and purely data-driven methods. The contributed formalization of place allows integration within GIS and maintains tangible properties associated with human understanding of place.

The proposed model and framework enable a better representation of the context that people assign to place than existing methods, such as gazetteers. In addition, they avoid limitations derived from subjectivity, as is the case with other models of place and interpretability, compared to purely data-driven approaches. These benefits are illustrated through the example of a function-based query to locate all shopping areas in Greater London. Answering this query through our framework allows capturing not only regions that are explicitly designated as shopping malls, but also those places that support similar functions. An additional side-result of the construction of a place from its components is the approximation of its spatial extent. This represents a minimum area in which the existence of a functional space is certain.

Future research directions include (a) automating the extraction of function-related information and the corresponding spatial configuration that enables them; (b) extending the composition model to support weighted composition rules that are not confined by the duality of logical values; (c) extending the model to allow place types to be described by multiple design patterns or patterns that depend on temporal dimensions; (d) extending the evaluation by consulting specialists, conducting surveys, or applying data-driven approaches; (e) exploring a fuzzy spatial representation of place that describes spatial extents using layers of certainty.

## References

[CIT0001] Alazzawi, A. N., Abdelmoty, A. I., & Jones, C. B. (2012). What can I do there? Towards the automatic discovery of place-related services and activities. *International Journal of Geographical Information Science*, 26(2), 345–364. doi:10.1080/13658816.2011.595954

[CIT0002] Couclelis, H. (1992). People manipulate objects (but cultivate fields): Beyond the raster-vector debate in GIS. In A. U. Frank, I. Campari, & U. Formentini (Eds.), *Theories and methods of spatio-temporal reasoning in geographic space* (pp. 65–77). Berlin: Springer.

[CIT0003] Couclelis, H. (2010). Ontologies of geographic information. *International Journal of Geographical Information Science*, 24(12), 1785–1809. doi:10.1080/13658816.2010.484392

[CIT0004] Curry, M. R. (1996). *The work in the world: Geographical practice and the written word*. Minneapolis, MN: University of Minnesota Press.

[CIT0005] Egenhofer, M. J., & Franzosa, R. D. (1991). Point-set topological spatial relations. *International Journal of Geographical Information System*, 5(2), 161–174. doi:10.1080/02693799108927841

[CIT0006] Fensel, D. (2001). *Ontologies*. Berlin: Springer.

[CIT0007] Frank, A. U. (1992a). Qualitative spatial reasoning about distances and directions in geographic space. *Journal of Visual Languages & Computing*, 3(4), 343–371. doi:10.1016/1045-926x(92)90007-9

[CIT0008] Frank, A. U. (1992b). Spatial concepts, geometric data models, and geometric data structures. *Computers & Geosciences*, 18(4), 409–417. doi:10.1016/0098-3004(92)90070-8

[CIT0009] Gao, S., Janowicz, K., & Couclelis, H. (2017). Extracting urban functional regions from points of interest and human activities on location-based social networks. *Transactions in GIS*, 21(3), 446–467. doi:10.1111/tgis.12289

[CIT0010] Gao, S., Janowicz, K., Montello, D. R., Hu, Y., Yang, J.-A., McKenzie, G., & Yan, B. (2017). A data-synthesis-driven method for detecting and extracting vague cognitive regions. *International Journal of Geographical Information Science*, 31(6), 1245–1271. doi:10.1080/13658816.2016.1273357

[CIT0011] Goodchild, M. F., & Hill, L. L. (2008). Introduction to digital gazetteer research. *International Journal of Geographical Information Science*, 22(10), 1039–1044. doi:10.1080/13658810701850497

[CIT0012] Graus, P. (2017). Place and placelessness revisited. *Urban Policy and Research*, 35(3), 369–371. doi:10.1080/08111146.2017.1329802

[CIT0013] Herrlich, H. (1974). A concept of nearness. *General Topology and Its Applications*, 4(3), 191–212. doi:10.1016/0016-660x(74)90021-x

[CIT0014] Janowicz, K., & Keßler, C. (2008). The role of ontology in improving gazetteer interaction. *International Journal of Geographical Information Science*, 22(10), 1129–1157. doi:10.1080/13658810701851461

[CIT0015] Jordan, T., Raubal, M., Gartrell, B., & Egenhofer, M. (1998). An affordance-based model of place in GIS. In T. K. Poiker & N. R. Chrisman (Eds.), *Proceedings of the 8th international symposium on spatial data handling* (pp. 98–109). Burnaby, B.C.: International Geographical Union, Geographic Information Science Study Group.

[CIT0016] Khan, A., Vasardani, M., & Winter, S. (2013). Extracting spatial information from place descriptions. In S. Scheider, B. Adams, K. Janowicz, M. Vasardani, & S. Winter (Eds.), *Proceedings of the first ACM SIGSPATIAL international workshop on computational models of place* (pp. 62–69). New York: ACM. doi:10.1145/2534848.2534857

[CIT0017] Kuhn, W. (2001). Ontologies in support of activities in geographical space. *International Journal of Geographical Information Science*, 15(7), 613–631. doi:10.1080/13658810110061180

[CIT0018] Montello, D. R., Friedman, A., & Phillips, D. W. (2014). Vague cognitive regions in geography and geographic information science. *International Journal of Geographical Information Science*, 28(9), 1802–1820. doi:10.1080/13658816.2014.900178

[CIT0019] Montello, D. R., Goodchild, M. F., Gottsegen, J., & Fohl, P. (2003). Where’s downtown?: Behavioral methods for determining referents of vague spatial queries. *Spatial Cognition & Computation*, 3(2–3), 185–204. doi:10.1080/13875868.2003.9683761

[CIT0020] Papadakis, E., Resch, B., & Blaschke, T. (2016). A function-based model of place. *International Conference on GIScience Short Paper Proceedings*, 1, 1. doi:10.21433/b3119z90g3zd PMC5826630 29492479

[CIT0021] Sanders, R. A., & Porter, P. W. (2010). Shape in revealed mental maps. *Annals of the Association of American Geographers*, 64(2), 258–267. doi:10.1111/j.1467-8306.1974.tb00975.x

[CIT0022] Scheider, S., & Janowicz, K. (2014). Place reference systems. *Applied Ontology*, 9(2), 97–127. doi:10.3233/AO-140134

[CIT0023] Scheider, S., & Purves, R. (2013). Semantic place localization from narratives. In S. Scheider, B. Adams, K. Janowicz, M. Vasardani, & S. Winter (Eds.), *Proceedings of the first ACM SIGSPATIAL international workshop on computational models of place* (pp. 16–19). New York: ACM. doi:10.1145/2534848.2534858

[CIT0024] Smith, B. (2001). Fiat objects. *Topoi*, 20(2), 131–148. doi:10.1023/A:1017948522031.

[CIT0025] Tuan, Y.-F. (1977). *Space and place: The perspective of experience*. Minneapolis, MN: University of Minnesota Press.

[CIT0026] Tuan, Y.-F. (1979). Space and place: Humanistic perspective. In S. Gale & G. Olsson (Eds.) *Philosophy in geography* (pp. 387–427). Dordrecht, Netherlands: Springer. doi:10.1007/978-94-009-9394-5. 19.

[CIT0027] Tversky, B. (2003). Structures of mental spaces: How people think about space. *Environment and Behavior*, 35(1), 66–80. doi:10.1177/0013916502238865

[CIT0028] Vasardani, M., Timpf, S., Winter, S., & Tomko, M. (2013). From descriptions to depictions: A conceptual framework. In T. Tenbrink, J. Stell, A. Galton, & Z. Wood (Eds.), *Spatial information theory* (pp. 299–319). Cham, Switzerland: Springer.

[CIT0029] Vasardani, M., Tomko, M., & Winter, S. (2016). The cognitive aspect of place properties. *International Conference on GIScience Short Paper Proceedings*, 1, 1. doi:10.21433/b3116928w12f PMC5826630 29492479

[CIT0030] Vasardani, M., Winter, S., & Richter, K.-F. (2013). Locating place names from place descriptions. *International Journal of Geographical Information Science*, 27(12), 2509–2532. doi:10.1080/13658816.2013.785550

[CIT0031] Vögele, T., Schlieder, C., & Visser, U. (2003). Intuitive modelling of place name regions for spatial information retrieval. In W. Kuhn, M. F. Worboys, & S. Timpf (Eds.), *Spatial information theory. Foundations of geographic information science* (pp. 239–252). Berlin: Springer.

[CIT0032] Winter, S., & Freksa, C. (2012). Approaching the notion of place by contrast. *Journal of Spatial Information Science*, 2012(5), 31–50. doi:10.5311/josis.2012.5.90

